# How Will the Global Food Landscape Accommodate Developing Countries’ Dietary Change under Urbanization?

**DOI:** 10.3390/foods11223598

**Published:** 2022-11-11

**Authors:** Yali Zhang, Saiya Li, Lu Jin, Feng Wu

**Affiliations:** 1 Key Laboratory of Land Surface Pattern and Simulation, Institute of Geographic Sciences and Natural Resources Research, Chinese Academy of Sciences, Beijing 100101, China; 2 College of Resources and Environment, University of Chinese Academy of Sciences, Beijing 100101, China; 3 School of Applied Economics, Renmin University of China, Beijing 100872, China

**Keywords:** diet, urbanization, food landscape, developing countries, GTAP

## Abstract

There has been a growing awareness of the dietary shift from traditional staples to animal-derived foods during the urbanization of developing countries. Less discussed is how the global food landscape will accommodate such changes in diet. Our study aims to use the GTAP (Global Trade Analysis Project) model to predict the future food landscape based on the dietary shift in developing countries, represented by China, India, Bangladesh, and Myanmar, under a 2030 urbanization scenario. The results show that the average global outputs of fish, meat, and dairy products increase by 0.26–2.85%, along with an expansion in their trade volume by 2.10–13.95%, by 2030. To ensure that dietary changes can be met in developing countries, Asia and America need to strengthen their positions with respect to global food production share, while Africa is developing to become a non-negligible growing force. Accordingly, globalized food trade is characterized by a centralized export and, conversely, by a decentralized import, clearly indicating an expanding net-import tendency in populous developing countries. These findings highlight the adaptation scheme of global food production and trade patterns under a 2030 urbanization scenario, as urbanization accelerates dietary change in developing countries.

## 1. Introduction

Urban sprawl triggers interlinkages in the food landscape through accelerations in dietary shifts towards food from animal sources [1]. On the one hand, considering an additional supply of meat, milk, and eggs, the necessity of production expansion has placed higher requirements on the utility of agricultural resources that becoming increasingly scarce (e.g., water and land) and sustainable modes of production [2, 3, 4]. On the other hand, the increasing share of animal-source food imports in net-importing countries raises concerns regarding the distortion of the international trade balance. These challenges may become more serious, as the urbanization rate is expected to increase inevitably by 16% on average in the next four decades [5]. This number is far greater in developing countries, such as China, India, Bangladesh, and Myanmar. Thus, it is critical to demonstrate the options for adaptation in the global food landscape under future dietary change in developing countries.

Dietary shifts occurring under the condition of urbanization are the primary progenitor of changes in the food landscape on the production side. This has mainly been observed in developing countries in Asia, where the sectors of the food system are integrated in mutually causal ways, rather than existing piecemeal and independently [6]. The changes in consumption directly impact production, from the perspective of both aggregate supply and production distribution. Coyle et al. [7] first applied the Global Trade Analysis Project (GTAP) model to point out that the preference for food from animal sources has contributed to its expanded production. Kastner et al. [8] analyzed the development of the global food supply and found a subsequent increasing or decreasing trend with changing demand. Studies have further projected future production patterns based on these regulations. Rosegrant et al. [2] used the IMPACT model to assess global agricultural supply, and reported a growing trend in the production of meat and milk. Kobayashi et al. [3] predicted the largest expansion of fish production to be in India in 2030, while the fastest-growing demand would be in China.

The food landscape has also experienced an evolution with respect to international trade as a result of the dietary transition being driven by urbanization. Countries with production surpluses are potentially developing their comparative advantages and becoming exporters, whereas regions in which domestic supply is not able to meet demand are reliant on imports to make up this gap in quantity. Coyle et al. [7] observed that those regions of China, ASEAN, and Japan that have undergone a dietary shift have generally seen the largest change in their trade structure. Smith et al. [9] explored global import penetration rates for staple food and animal products over the period from 1995 to 2018 and found increases in imports of meat and milk products by 25% and 15.7%, respectively. Ma et al. [10] applied the NUFER model and demonstrated an increase in food imports by 10% in China in 2030. In the future, developing countries will probably expand their import volume of livestock-derived food. 

Previous studies have used evidence from one region or from highly connected markets in nearby countries to understand changes in food production and trade patterns occurring in conjunction with dietary shifts. However, the comprehensive picture of the global food landscape with the acceleration in dietary shifts has still been little studied [1]. In addition, most studies have concentrated on a single food category instead of examining various food items. Our study aims to give some perspective on the international food market by considering all-round food items. This study focuses on dietary changes under urbanization in developing countries, which are represented by China, India, Bangladesh, and Myanmar. These countries were selected as they consume approximately 40% of the world’s total food, due to their high populations, and they are expected to contribute a large proportion to newly added urban dwellers, globally [11], as well as undergoing a significant adjustment in consumption patterns [12,13]. Our study is further indispensable in terms of supplying options by means of which the global food landscape might adapt, in order to be able to endure the dietary shifts occurring in developing countries. 

The remainder of this paper is organized as follows. In Section 2, we quantify the change in demand for major food items, including cereals, meat, fish, dairy products, vegetables, and fruits, in China, India, Bangladesh, and Myanmar under a 2030 urbanization scenario. Using the GTAP model, the projected global food landscape is presented with respect to outputs, fluctuating price, and international trade in Section 3. Section 4 presents a discussion, along with policy implications, of our results, as well as the strengths and limitations of the study. We then conclude by analyzing the implications of our findings in Section 5.

## 2. Data and Methods

### 2.1. GTAP Modeling

As a well-organized multi-regional, multi-sector computable economic equilibrium model, GTAP provides a mainstream tool for the dynamic analysis of trade policies, environment, population, energy, and climate change [14,15,16]. GTAP, a comparative static analysis model, flexibly analyzes the impact for agricultural commodities on the production, price, supply, and demand of primary factors (capital, land, natural resources, skilled labor, and unskilled labor), import and export trade, and social welfare level. The model assumes a completely competitive market, constant returns to scale, and market clearing. It is theoretically assumed that producers reach profit maximization, while it is also presumed that utility maximization is achieved for consumers. The balance of aggregate supply and demand determines the values of endogenous variables, namely price, wages, and land rents. 

GTAP incorporates complex but comprehensive modeling analysis. The production structure inside GTAP is constructed from a set of nested CES (constant elasticity of substitution) functions. Two aggregate components, intermediate demand and value-added, form the top-level nest. They are resolved and then recombined into components in the second level, and thus the demand for individual intermediate goods and primary factors are divided. Incomplete substitution between domestic products and imported commodities is accepted in the final nest. On the consumption side, the constant differences in elasticities in the implicit additive expenditure function are representative of a private household’s preferences, while the Cobb–Douglas function describes government consumption. The Leontief utility function presents a sub-unity for investment expenditure, which is subsequently decomposed into domestic demand and imported goods using a CES sub-utility preference function.

Our study aims to represent the global landscape in the context of dietary change in developing countries under a 2030 urbanization scenario. Here, changes in the dietary structure of developing countries (represented by Bangladesh, China, India, and Myanmar) are entered into GTAP as a shock factor, while the dietary structure in the rest of world is assumed to remain constant. The GTAP model is then used to estimate the output, price, export, and import for 40 countries/regions, which are then combined into Australia, Africa, America, Europe, West Asia, and East Asia. The 5 primary factors are aggregated into three categories (land, capital, and labor), and the production sectors referred to as food commodities are the main focus. The detailed data were obtained from the latest version (v10) of the GTAP database using a base year of 2014. 

### 2.2. Urbanization Scenario Setting

Urbanization is accelerating in developing countries as an inevitable trend due to natural population growth, the reclassification of previously rural land into urban land, and rural–urban migration [17]. The proportion of urban to total area nearly doubled between 1965 and 2014 in Bangladesh, China, India, and Myanmar [5]. This ongoing tendency will continue, resulting in a higher urbanization rate in 2030, as predicted by World Bank, increasing by 12.10 percentage points in Bangladesh (33.50–45.60%), 16.30 points in China (54.30–70.60%), and 7.70 and 5.30 points in India (32.40–40.10%) and Myanmar (29.70–35.00%), respectively. 

The difference between urban and rural per capita consumption are first estimated in order to forecast changes in food consumption in 2030. To indicate the impact brought about by the progress of rapid urbanization, a forecast of changes in food consumption (*fcc*) is established as an indicator to evaluate dietary change in the form of a percentage value:

(1) $$fcc=\frac{dpc\times cur\times pop}{tfc}$$

where *dpc* is per capita consumption in the urban area minus that in the rural area, and *cur* measures the development of urbanization, which is equal to the urbanization rate in 2030 predicted by the World Bank minus that in 2014. The forecast of changes in food consumption (*fcc*) in 2030 is then calculated by multiplying *dpc* with *cur* and population (*pop*) divided by total food consumption (*tfc*) (Table 1). 

## 3. Results

### 3.1. Global Food Production Patterns Driven by Developing Countries’ Dietary Changes 

**Figure 1:**
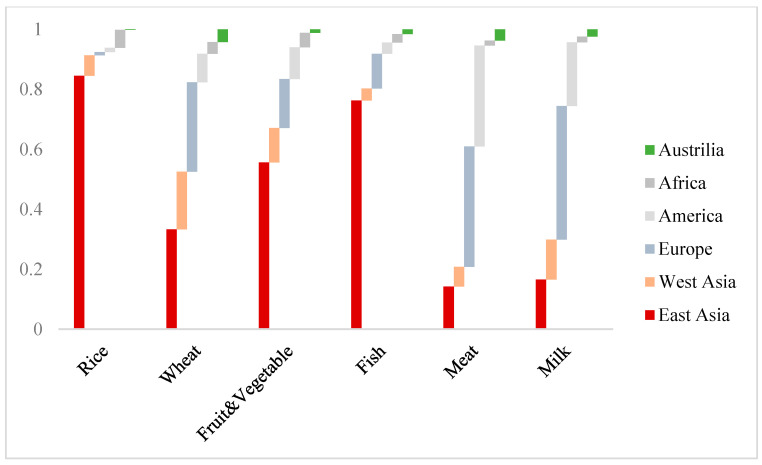
Percentage contribution of different regions to global aggregate supply in 2030.

Under the 2030 urbanization scenario, the demand-oriented food outputs are forecast to share the same changing direction as that of dietary change (Figure 1). Among all categories, fish, meat, and milk production show the most obvious growing trend. The rate of increase of fish reaches 2.85%, which is the highest, and approximately 48 times that of cereals (which increases by 0.06%) and 6 times that of fruits and vegetables (which increases by 0.49% in total). Dairy and meat products are second and third, with their output expanding by 1.17% and 0.62%, respectively. Meanwhile, there is a decrease in rice amounting to 0.15% due to reduced consumer demand. 

The global food production pattern remains relatively stable. Asia contributes more than half of the total output in almost every food category. China and India, the most populous developing countries, maintain their status in the production of rice (accounting for 88.5% of the total) and wheat (accounting for 54.5% of the total). South Asian regions typified by Myanmar, Indonesia, and Bangladesh are in the second tier of paddy rice, and Western Asian countries, represented by Pakistan and Turkey, are expected to account for a share 0.44% greater in the production of wheat. Asian developing countries also have an advantage in fishing and planting fruits and vegetables, accounting for shares of 80.30% and 67.10%, respectively. Besides Asia, it is indicated that Europe and America will maintain their comparative advantage with regard to meat, raw milk, and dairy products, along with animal feed, including corns, soybeans, and other crops. Europe and America are projected to make up more than half of global corn and soybean production, laying the best foundation for breeding livestock (accounting for 46.24%) and making raw milk (63.10% of the total). The production of animal products in Western developed countries thus stays ahead. Europe produces 40.13% of total meat and 44.54% of total dairy products, followed by America, at 33.65% and 21.26%, respectively. 

**Figure 2:**
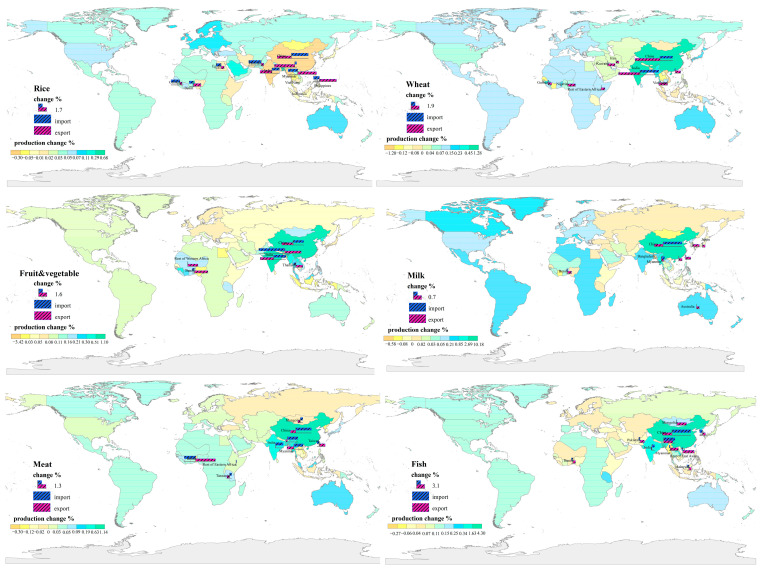
The geographical distribution of production and trade growth rates. Six main food categories, namely rice, wheat, fruits & vegetables, milk, meat, and fish, are presented. The changes in production volume of each category in different regions are indicated by background colors. The most apparent changes in import and export volumes were chosen and are shown in the histograms.

The growth rate of grain production ranges from −3.42% to 10.18% in Asian regions in 2030, when compared with the 2014 baseline scenario (Figure 2). Developing countries in West and Central Asia show a more significant increase in cereals than that in East and Southeast Asia. Among all of the regions, Bangladesh is projected to have the highest rate of increase, at 0.67%, for paddy rice, followed by Benin, Saudi Arabia, and the UAE, at 0.63%, 0.29%, and 0.22%, respectively. However, China, India, and Myanmar are projected to reduce their rice production volume by 0.30–0.36%. Wheat and crop production volumes have almost the same sign, except for that in China. China produces 1.04% more wheat, second only to India (which increases by 1.26%), showing the opposite direction of change to other East and Southeast Asian countries (which decrease by approximately 0.19% on average). Crops grown in China are indicated to increase by 0.20%, which is 10 times higher than the increase in Central Asian regions, while in other East Asian countries, represented by Myanmar and Thailand, the rates of decrease reach 0.18% and 0.24%, respectively. Besides Asia, African countries are expected to become another growing force, not only in grain, but also in terms of the production volumes of fruits and vegetables. In 2030, the growth rates of Africa’s paddy rice and wheat reach 1.10% and 0.78%, respectively, which is approximately 10 times greater than Europe and 20 times greater than America. Cereal production volumes in Nigeria, Guinea, and Tanzania are the main factor supporting this. There is also an increase in fruit and vegetable production volumes, by 1.58%. Unlike improvements in yield brought about by higher productivity in Europe and America, agricultural growth in Africa is mainly due to the expansion of harvested areas [18]. 

### 3.2. Global Food Trade Patterns Driven by Developing Countries’ Dietary Changes

**Figure 3:**
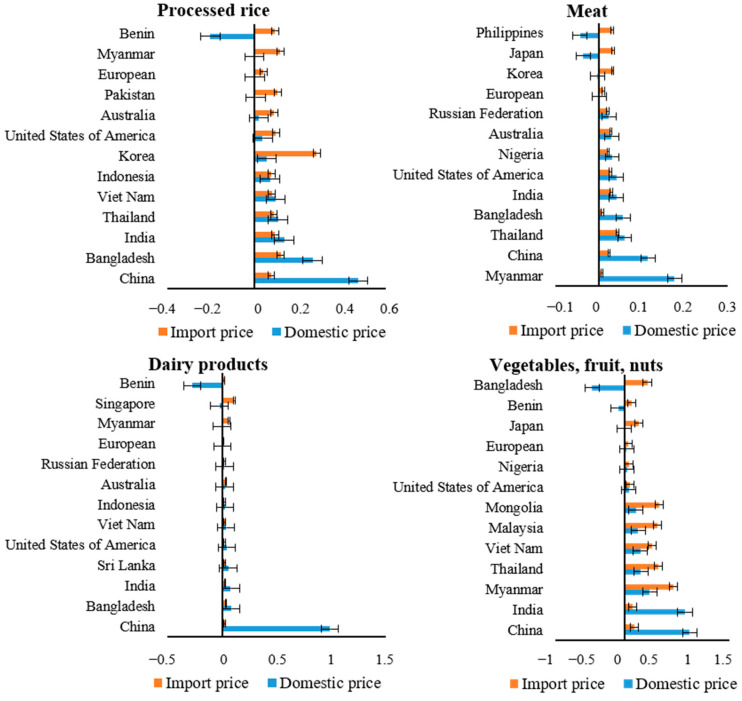
The most representative price fluctuations for four food categories in 2030.

The imbalance between the demand-side dietary shift and supply-side changes in food production greatly impact both domestic and import prices. From the perspective of domestic prices, East and Southeast Asian developing countries are projected to present higher internal price levels for most food items (Figure 3). Domestic food prices in China increase in fish (1.95% higher), milk and dairy products (0.99%), and fruits and vegetables (0.93%). The costs of cereals, meat, and oil crop also reveal a significant increase (in the range from 0.51 to 0.69%). The overall price increase in China is mainly caused by higher factor prices. Further urbanization will lead to a decrease in cultivated land, and higher land cost as a result (going up by 4.31%), along with shortages of agricultural labor and the related wage increases. There also exists a giant demand-supply gap whereby the increase in output is not able to keep up with the faster growth in demand. The regulations governing the increasing domestic prices in China apply to other East and Southeast Asian regions. India sells wheat, sugar cane, oil crop, vegetables, and fruits at prices more than 0.63% higher. In Bangladesh, price rises mainly occur in fish (0.88%), sugar (0.66%), and paddy rice (0.47%). Countries increase their domestic price by an average of about 0.10% in East and Southeast Asia, represented by Vietnam, Thailand, Singapore, Malaysia, and Mongolia. In contrast, internal food price levels in Europe, Africa, and Western and Central Asia hold steady in the projection (at a rate of increase of no more than 0.03%). Noticeably, the African country Benin is forecast to present a significant decrease in the price of meat and dairy products, by 0.30% and 0.27%, respectively, as a result of the benefits of its stable land price and reduced capital cost. 

On the other hand, most countries’ import prices do not fluctuate with their domestic prices. Import prices in populous Asian countries remain stable, which is in stark contrast to their skyrocketing internal prices. China and India, as representatives, only increase the price of their import cereals and livestock-derived food by a maximum of 0.05%. In comparison, the growth rate of the price of imported fruits and vegetables in other South and Southeast Asian regions surpasses that of products sold at the local market (0.70% versus 0.36% in Myanmar, 0.32% versus –0.47% in Bangladesh, 0.47% versus 0.09% in Indonesia, 0.48% versus 0.12% in the Philippines, and 0.48% versus 0.23% in Thailand). The domenstic price advantage theoretically motivates fruit and vegetable export in these countries. Several Western Asian countries enjoy internal price superiority with respect to paddy rice and wheat, namely Pakistan, Saudi Arabia, and Iran. The difference between the growth rates of domestic and import cereals prices in these countries reaches 0.15–0.60%. The same difference is seen in Africa. A more apparent increase in import price emerges than in domestic prices in most of the African countries, especially in Benin. Predictably, the noticeable increasing speed of the domestic and import price change between regions will influence their international trade patterns.

The globalization of food trade promotes deepening division, resulting in the concentration of major food production in advantageous regions. Global export patterns become centralized (Figure 2). Of the paddy rice exported in 2030, Asian and American countries account for 81.10% of the total. European countries are projected to have net wheat and corn export positions, comprising 60.58% and 41.73% of the global aggregate export volume. Similar to the distribution of livestock feed exports, animal-derived products (meat and dairy) are centered in America and Europe. China, Indonesia, and Europe account for 78.51% of exported fish. Other coastal Asian countries, namely Myanmar, Bangladesh, Vietnam, and the Philippines, present rates of increase in terms of fish exports that are above the global average (ranging from 1.26% to 3.13%). Increased exports in Africa mainly depend on fruits and vegetables (more than 1%, double the global average). Due to having little competitive advantage when it comes to food processing, African countries increase their export share of unprocessed materials, including raw milk and livestock. Benin, the main exporter in Africa, takes full advantage of competitive domestic prices and expands its exports of milk (2.89%), meat (2.65%), and fruits and vegetables (2.64%).

In contrast to centralized export patterns, importers are relatively scattered around the world. The import concentration is projected to drop, as most of the countries expand their imports: corn in East and Southeast Asia (namely Japan, Korea, Vietnam, and Malaysia), grains in Africa (Tanzania and Cote d’Ivoire), sugar in Europe, and fish and meat products in America. Among all of the importers, populous Asian countries rely the most on the trade of food. The increase in the import volume of livestock-derived food surpasses that of other food items in those countries. In the case of China, the demand for fish and dairy products is projected to be considerably high in external dependence (increase by 6.29% and 13.95%) compared to that of rice and wheat (increase by 3.06% and 2.53%). A contributing factor to the slower pace of meat import growth (2.10%) could be that per capita consumption has already increased substantially in the last decade, leaving narrow scope for further increase. In addition to China, growth rates for meat, milk, and sugar in Bangladesh, Myanmar and India are expected to be quite high in response to the dietary structural shift. Intra-Asian trade becomes more frequent. Trade volume to China increases by 3.36% for paddy rice from Myanmar, by 1.60–2.33% for milk and dairy products from India and Myanmar, and by 3.20% or more for fish from Bangladesh, Thailand, and Indonesia. The acceleration of the Intra-Asian connection corroborates results reported in previous studies [19].

## 4. Discussion and Policy Implications

The efficiency of food production patterns is now on the agenda in order to be able to accommodate the inevitable trend of production expansion. Our predictions indicate that populous developing Asian and underdeveloped African countries will be the greatest potential force in the global trend of output expansion. These regions are relatively less experienced with highly intensive agricultural technology and regulatory frameworks [20,21]. During the past three to four decades, only modest growth has been presented by some Asian countries, and there has been no change demonstrated by Africa in terms of agricultural yield [22]. Unless the efficiency of resource use is improved, the burden on agricultural resources (e.g., land and water) will continuously and critically add up in an unsustainable way. Substantial technical improvements in production efficiency, including cultivating improved maize seeds and perfecting agricultural infrastructure, are necessary in order to achieve increased and improved production in developing countries [10]. The transition to larger-scale and more intensive production patterns has been proven to provide better conditions for output expansion [23]. Increased investment and agricultural subsidies can also work to improve the food supply [24].

There will be a surge in the production of livestock-derived food, driven by a convergence in its demand. Livestock farming is characterized by lower productivity and varying intensification level, while requiring greater investment, and producing greater emissions and pollution [25,26,27], thereby exacerbating the risk of unsustainable expansion, such as in the form of increasing animal stocks without the use of intensive production systems [28] in order to meet the expected food demand. The coordination of food supply and environmental capacity through the combination of better grazing management systems, precious and efficient livestock breeding patterns, and integrated production poses a challenge [10,29,30]. The expansion of livestock farming also leads to higher requirements of animal feed. It is necessary for a larger amount of maize and soybeans to be produced as feed, instead of as rations, implying a structural change in crop varieties [31]. Approaches to various improvements and forage crop cultivation, as well as investment, are fundamental in supporting the expansion of meat and dairy production.

Challenges regarding import reliance arise, as global trade is indicated to be increasingly crucial to the aggregate food supply. Our results indicate a growing trend in food import in populous developing countries and low-income regions, which is in line with the more defined core-periphery network structure predicted by Dong et al. [32]. Developing countries with population prosperity will shift their approach to become net-importers, whereas developed regions, Europe and America for instance, will firmly position themselves in food export by employing leading agricultural technology and management systems. The growing reliance on imports threatens food security in developing countries. For example, China has already increased its import volume of rice, wheat, and soybeans in recent decades [19], and is emerging as a burgeoning importer of meat and dairy products. Our results indicate a tendency to rely continuously on the global food market in 2030, revealing a reduction in exports, but a comprehensive rise in imported commodities. This extremely high external dependency of the food system distorts the international trade balance [33]. The lack of initiative in ensuring supply availability threatens net-importers [34], since food export from trade partners could be easily hampered by output scarcity caused by trade policies related to political factors, along with the phenomenon whereby the higher food reliance will result in more severe impacts of local price fluctuations. Harsh but necessary improvements in domestic supply and appropriate trade policy are considered key to reducing risks from the international food market and guaranteeing food security.

This study aims to fill a knowledge gap in the understanding of the adaptation of the global food landscape to changing urbanized diets. The study focuses on highlighting the role of dietary change in developing countries in global food production and trade. The variety of demand-side foods adds a complementary perspective to the existing body of research by considering a single food category. Given the fact that the model results are based on the simplifying assumption of constant diets in rest of world, except for China, India, Bangladesh, and Myanmar, this may not reflect the real-life food landscape. Another potential limitation of the model simulations is that the scenario setting does not take into account complex emergencies such as pandemics and wars. These emergencies could disrupt supply chains and thus sever trade links. In addition, the model predicts greater exports in Europe/America, as they have a technological advantage, while technologies change in less developed countries may partly catch up in the future.

## 5. Conclusions

The GTAP modeling framework allows the integrated prediction of the food landscape in 2030. The growing need to put forward broad-based adjustment options for the food sector that achieve multiple objectives with respect to food security, within the context of accelerated urbanization and Sustainable Development Goals (SDGs), motivated this study. The changes in dietary structure in China, India, Bangladesh, and Myanmar—four developing countries characterized by rapid urban expansion—are initially projected as a fundamental shock, in order to interact them with global food supply, price, and trade patterns. The evidence presented in this paper confirms a differentiation in the developing trends of consumption, production, and transaction between diverse countries and food categories. The results demonstrate that the most apparent increase will be in the output of fish, meat, and dairy products, while there will be a growing reliance on food imports from developing countries. The change in the future food landscape underscores the importance of discussing countermeasures to solve the dilemma presented by rising demand in the context of issues regarding agricultural resource utility and international trade stability.

Developing countries will need to be prioritized within a more global agenda in order to achieve food security. The growing demand for livestock-derived food will lead to a surge in production growth and import reliance in developing countries, represented by China and India. To address these challenges, these countries need to focus on intervention options that improve the capacity of sustainable production, e.g., by applying new agricultural technologies in order to achieve higher resource use, and distribution policies to channel food to target areas. In addition, optimizing the overseas distribution of various kinds of food is of core significance in guaranteeing global food sources. Regions with abundant agricultural resources and advanced technologies should maximize their advantages. Investments that support self-sufficiency in developing countries will also need to embed strategies that promote trade, while also managing global food security issues that could arise.

## Figures and Tables

**Table 1:** Projections of changes in food consumption for selected food categories in Bangladesh, China, India, and Myanmar in 2030 under the urbanization scenario.

Item	Bangladesh (%)	India (%)	China (%)	Myanmar (%)
Cereal grains nec	−2.81	−4.03	−6.76	−0.28
Vegetables, fruit, nuts	−3.65	2.08	4.41	0.78
Oil seeds	0.41	3.71	5.37	0.14
Animal products nec	0.00	2.35	5.07	1.68
Fishing	5.00	1.66	11.68	0.00
Meat: cattle, sheep, goats, horse	5.73	3.02	6.85	1.79
Meat products nec	0.00	3.02	1.34	1.79
Vegetable oils and fats	0.00	0.00	1.93	0.78
Dairy products	−0.44	1.42	15.40	6.38
Processed rice	1.32	0.00	0.00	−0.53
Sugar	3.04	3.87	0.00	−2.98
Food products nec	−2.99	3.27	0.00	0.00

Sources: Household Income and Expenditure Survey (2016); China Statistical Yearbook (2014); National Family Health Survey (NFHS-4, 2015); Household Dietary Patterns and the Cost of a Nutritious Diet in Myanmar (2012).

## Data Availability

The date are available from the corresponding author.

## References

[1] Hatab A.A., Cavinato M.E.R., Lindemer A., Lagerkvist C.-J. (2019). Urban sprawl, food security and agricultural systems in developing countries: A systematic review of the literature. Cities.

[2] Rosegrant M.W., Tokgoz S., Bhandary P. (2013). The new normal? A tighter global agricultural supply and demand relation and its implications for food security. Am. J. Agric. Econ..

[3] Kobayashi M., Uesugi S., Hikosaka R., Aikawa R. (2015). Relationship between professional experience as a practicing dietitian and lifestyle and dietary habits among graduates of a department of food science and nutrition. Nutr. Food Sci..

[4] Geyik O., Hadjikakou M., Bryan B.A. (2020). Spatiotemporal trends in adequacy of dietary nutrient production and food sources. Glob. Food Secur..

[5] United Nations (2014). World Urbanization Prospects, 2014 Revision.

[6] Reardon T., Timmer C.P. (2014). Five inter-linked transformations in the Asian agrifood economy: Food security implications. Glob. Food Secur..

[7] Coyle W., Emhlhar M., Hertel T.W., Wang Z., Yu W. (1998). Understanding the determinants of structural change in world food markets. Am. J. Agric. Econ..

[8] Kastner T., Rivas M.J.I., Koch W., Nonhebel S. (2012). Global changes in diets and the consequences for land requirements for food. Proc. Natl. Acad. Sci. USA.

[9] Smith V.H., Glauber J.W. (2020). Trade, policy, and food security. Agric. Econ..

[10] Ma L., Bai Z.H., Ma W.Q., Guo M.C., Jiang R.F., Liu J.G., Oenema O., Velthof G.L., Whitmore A.P., Crawford J. (2019). Exploring future food provision scenarios for China. Environ. Sci. Technol..

[11] United Nations (2014). World Population Prospects, 2014 Revision.

[12] Seto K.C., Ramankutty N. (2016). Hidden linkages between urbanization and food systems. Science.

[13] D’Amour C.B., Pandey B., Reba M., Ahmad S., Creutzig F., Seto K.C. (2020). Urbanization, processed foods, and eating out in India. Glob. Food Secur..

[14] Schandl H., Hatfield-Dodds S., Wiedmann T., Geschke A., Cai Y., West J., Newth D., Baynes T., Lenzen M., Owen A. (2016). Decoupling global environmental pressure and economic growth: Scenarios for energy use, materials use and carbon emissions. J. Clean. Prod..

[15] Xie W., Ali T., Cui Q., Huang J. (2017). Economic impacts of commercializing insect-resistant GM maize in China. China Agric. Econ. Rev..

[16] Ali T., Huang J.K., Wang J.X., Xie W. (2017). Global footprints of water and land resources through China’s food trade. Glob. Food Secur..

[17] McGranahan D.A. (2014). Ecologies of scale: Multifunctionality connects conservation and agriculture across fields, farms, and landscapes. Land.

[18] Brink L. (2009). WTO Constraints on domestic support in agriculture: Past future. Can. J. Agric. Econ..

[19] Feng Z.M., Xiao C.W., Peng L. (2017). Spatio-temporal pattern changes of cereal production and trade in China-ASEAN free trade area. J. Nat. Resour..

[20] Ma L., Wang F., Zhang W., Ma W., Velthof G., Qin W., Oenema O., Zhang F. (2013). Environmental assessment of management options for nutrient flows in the food chain in China. Environ. Sci. Technol..

[21] West C.T., Somé A., Nebié E.K. (2014). Famines are a thing of the past: Food security trends in Northern Burkina Faso. Hum. Organ..

[22] Enahoro D., Mason-D’Croz D., Mul M., Rich K.M., Robinson T.P., Thornton P., Staal S.S. (2019). Supporting sustainable expansion of livestock production in South Asia and Sub-Saharan Africa: Scenario analysis of investment options. Glob. Food Secur..

[23] Lu C.X., Liu A.M., Xiao Y., Liu X.J., Xie G.D., Cheng S.K. (2020). Changes in China’s grain production pattern and the effects of urbanization and dietary structure. J. Resour. Ecol..

[24] Law C., Green R., Kadiyala S., Shankar B., Knai C., Brown K.A., Dangour A.D., Cornelsen L. (2019). Purchase trends of processed foods and beverages in urban India. Glob. Food Secur..

[25] Gerber P., Chilonda P., Franceschini G., Menzi H. (2005). Geographical determinants and environmental implications of livestock production intensification in Asia. Bioresour. Technol..

[26] Nijdam D., Rood T., Westhoek H. (2012). The price of protein: Review of land use and carbon footprints from life cycle assessments of animal food products and their substitutes. Food Policy.

[27] Havlík P., Valin H., Herrero M., Obersteiner M., Schmid E., Rufino M.C., Mosnier A., Thornton P.K., Böttcher H., Conant R.T. (2014). Climate change mitigation through livestock system transitions. Proc. Natl. Acad. Sci. USA.

[28] Food and Agriculture Organization (FAO) (2015). FAOSTAT Statistics Database of the Food and Agricultural Organization of the United Nations (FAO).

[29] Kolver E.S., Muller L.D. (1998). Performance and nutrient intake of high producing Holstein cows consuming pasture or a total mixed ration. J. Dairy Sci..

[30] Huan-Niemi E., Niemi J., Niemi J. (2010). Global Food Production under Alternative Scenarios. Int. Food Agribus. Manag. Rev..

[31] Zhang X., Liu Y., Liu Y., Cui Q., Yang L.Y., Hu X.H., Guo J.L., Zhang J.Z., Yang S.X. (2019). Impacts of climate change on self-sufficiency of rice in China: A CGE-model-based evidence with alternative regional feedback mechanisms. J. Clean. Prod..

[32] Dong C., Yin Q.J., Lane K.J., Yan Z.J., Shi T.Y., Liu Y., Bell M.L. (2018). Competition and transmission evolution of global food trade: A case study of wheat. Physica A.

[33] Liu Q., Liu X.L., Wang S.Y. (2018). Estimating China’s food demand from 2020 to 2050 based on reasonable dietary pattern. Syst. Eng.-Theory Pract..

[34] Puma M.J., Bose S., Chon S.Y., Cook B.I. (2015). Assessing the evolving fragility of the global food system. Environ. Res. Lett..

